# Novel Evidence that Purinergic Signaling - Nlrp3 Inflammasome Axis Regulates Circadian Rhythm of Hematopoietic Stem/Progenitor Cells Circulation in Peripheral Blood

**DOI:** 10.1007/s12015-020-09953-0

**Published:** 2020-01-15

**Authors:** Mateusz Adamiak, Andrzej Ciechanowicz, Marta Skoda, Monika Cymer, Michal Tracz, Bing Xu, Mariusz Z. Ratajczak

**Affiliations:** 1 Department of Regenerative Medicine, Center for Preclinical Research and Technology, Medical University of Warsaw, Warsaw, Poland; 2 Institute of Veterinary Medicine, Department of Food Hygiene and Public Health Protection, Warsaw University of Life Sciences, Warsaw, Poland; 3 Department of Hematology, The First Affiliated Hospital of Xiamen University and Institute of Hematology of Xiamen University, Xiamen, People’s Republic of China; 4 Stem Cell Institute at James Graham Brown Cancer Center, University of Louisville, 500 S. Floyd Street, Rm. 107, Louisville, KY 40202 USA

**Keywords:** Diurnal rhythm, Circadian rhythm, Nlrp3 inflammasome, Extracellular ATP, Hematopoietic stem cells

## Abstract

We found that circadian changes in ATP level in peripheral blood (PB) activate the Nlrp3 inflammasome, which triggers diurnal release of hematopoietic stem/progenitor cells (HSPCs) from murine bone marrow (BM) into PB. Consistent with this finding, we observed circadian changes in expression of mRNA for Nlrp3 inflammasome-related genes, including Nlrp3, caspase 1, IL-1β, IL-18, gasdermin (GSDMD), HMGB1, and S100A9. Circadian release of HSPCs from BM into PB as well as expression of Nlrp3-associated genes was decreased in mice in which pannexin 1-mediated secretion of ATP was inhibited by the blocking peptide 10Panx and in animals exposed to the specific small-molecule inhibitor of the Nlrp3 inflammasome MCC950. In addition to HSPCs, a similar decrease in diurnal cell counts was observed for mesenchymal stromal cells (MSCs), endothelial progenitor cells (EPCs), and very small embryonic-like stem cells (VSELs). These results shed more light on the complexity of circadian regulation of HSPC release into PB, which is coordinated in a purinergic signaling-, innate immunity-dependent manner. Moreover, in addition to circadian changes in expression of the Nlrp3 inflammasome we also observed diurnal changes in expression of other inflammasomes, including Aim2, Nrp1a, and Nlrp1b.

## Introduction

Circadian rhythms are internal processes that regulate the sleep–wake cycle and repeat every 24 h. These processes are driven by a circadian clock and have been evolutionarily established in plants, fungi, bacteria, and animals, including mice and humans [1–5]. It is well known that this intrinsic circadian clock regulates all aspects of diurnal behavior and physiology, which includes melatonin secretion by the pineal gland, body temperature, and the plasma level of cortisol in mammals [5–8].

The number of circulating hematopoietic stem/progenitor cells (HSPCs) in peripheral blood (PB) follows a circadian rhythm pattern, with the peak occurring in the early morning hours and the nadir at night [3, 6, 9, 10]. In an elegant paper it was demonstrated that the timing of this peak can be explained by fluctuations in the tonus of the vegetative nervous system [4].

However, diurnal release of HSPCs from bone marrow (BM) into PB is also regulated by three evolutionarily ancient serum proteolytic cascades, the complement cascade (ComC), the coagulation cascade (CoaC), and the fibrinolytic cascade (FibC), whose activation is triggered in a circadian manner during deep-sleep hypoxia in the late-night hours [1, 8, 11–13]. This pattern of activation correlates with diurnal fluctuations in the level of the bioactive phosphosphingolipid sphingosine-1-phosphate in PB, which is a potent chemoattractant for HSPCs and mediates their egress from BM into the circulation [8, 14]. Recent evidence from our laboratories indicates that an important role in triggering stress-related or pharmacological mobilization of HSPCs is played by extracellular adenosine triphosphate (ATP), a crucial mediator of the purinergic signaling network [15–17]. ATP is released from activated or stressed cells, as seen during hypoxia or after administration of pro-mobilizing agents, and activates the Nlrp3 inflammasome protein complex in innate immunity cells by engaging the P2X7 purinergic receptor on the cell surface [17–21].

Supporting a crucial role for the Nlrp3 inflammasome complex in the mobilization of HSPCs, inhibition of this complex by the small-molecule inhibitor MCC950 impairs mobilization in response to administration of the pro-mobilizing cytokine granulocyte colony-stimulating factor (G-CSF) or the CXCR4 receptor antagonist AMD3100 [17]. Based on these observations, we hypothesized that deep-sleep hypoxia first releases ATP, which subsequently triggers the Nlrp3 inflammasome and in turn mobilizes HSPCs into PB in a circadian rhythm-dependent manner.

To address this intriguing question we measured the diurnal level of ATP in PB, evaluated changes in expression of mRNAs for Nlrp3 inflammasome-related genes, inhibited secretion of ATP by employing the pannexin 1 channel-blocking peptide 10Panx [21], and exposed mice to a small-molecule inhibitor of the Nlrp3 inflammasome, MCC950 [17, 22, 23]. Here for the first time we provide evidence that extracellular ATP-mediated purinergic signaling drives circadian release of HSPCs into PB in an Nlrp3 inflammasome-dependent manner.

## Materials and Methods

### Animals

Experiments were performed in C57BL/6 mice (Central Laboratory for Experimental Animals, Medical University of Warsaw, Poland or Jackson Laboratory, Bar Harbor, ME, USA). Mice, control (WT), treated with the NRLP3 inhibitor MCC950 (i.p., 25 mg/kg, twice a day for 2 weeks), or treated with the Panx-1 mimetic inhibitor 10Panx, which blocks pannexin 1 gap junctions (i.p., 4 days, 75 mg/kg), were accustomed to alternating periods of 12 h light and 12 h darkness. Light was turned on at 6 AM (ZT0), and the number of circulating white blood cells (WBCs), SKL cells, clonogenic CFU-GM progenitors, non-hematopoietic very small embryonic-like stems cells (VSELs), mesenchymal stromal cells (MSCs), and endothelial progenitor cells (EPCs) were measured at 7 AM (ZT1), 11 AM (ZT5), 7 PM (ZT13), and 3 AM (ZT21). At the same time points, the ATP level was evaluated by colorimetric assay in mouse plasma. Animal studies were approved by the Animal Care and Use Committee of the Warsaw Medical University (Warsaw, Poland) and University of Louisville (Louisville, KY, USA).

### Peripheral Blood Parameters

To obtain leukocyte counts, blood samples were collected from the retro-orbital plexus of mice into microvette EDTA-coated tubes (Sarstedt Inc., Newton, NC, USA) and run within 1 h of collection on a HemaVet 950 hematology analyzer (Drew Scientific Inc., Oxford, CT, USA). Additional plasma was collected for colorimetric assays. For SKL, CFU-GM, VSEL, MSC, and EPC analysis, blood was collected from the vena cava (with a 25-gauge needle and 1-ml syringe containing 250 U heparin) [15, 17].

### Enumeration of Colony-Forming Unit-Granulocyte/Macrophage (CFU-GM)

After PB red blood cell lysis (BD Pharm Lyse Buffer, San Jose, CA, USA), nucleated cells were washed and counted, and 1 × 106 cells were resuspended in RPMI-1640 culture medium (Corning Co, Corning, NY, USA) or in human methylcellulose base medium provided by the manufacturer (R&D Inc., Minneapolis, MN, USA), supplemented with murine GM-CSF (25 ng/ml) and IL-3 (10 ng/ml). Cultures were incubated for 7 days (37 °C, 95% humidity, and 5% CO2), at which time they were scored under an inverted microscope for the number of colonies. For evaluation of the number of circulating CFU-GM colonies the following formula was used: (number of white blood cells (WBCs) × number of CFU-GM colonies)/number of WBCs plated = number of CFU-GM per μl of PB [15, 17].

### Fluorescence-Activated Cell Sorting (FACS) Analysis of Circulating Stem Cells

For staining of SKL cells (Sca-1+ c-Kit+ Lin–), VSELs (Sca-1+ Lin– CD45–), MSCs (Lin– CD45– CD31– CD90+), and EPCs (Lin– CD45– CD31+) the following monoclonal antibodies were used: FITC–anti-CD117 (also known as c-Kit, clone 2B8; BioLegend, San Diego, CA, USA) and PE–Cy5–antimouse Ly-6 A/E (also known as Sca-1, clone D7; eBioscience, San Diego, CA, USA). All anti-mouse lineage marker antibodies, including anti-CD45R (also known as B220, clone RA3-6B2), anti-Ter-119 (clone TER-119), anti-CD11b (clone M1/70), anti-T cell receptor β (clone H57–597), anti-Gr-1 (clone RB6-8C5), anti-TCRγδ (clone GL3), and anti-CD45 (clone 30-F11), conjugated with PE; anti-CD31 (clone MEC 13.3), conjugated with APC; and anti-CD90.2 (clone 30-H12), conjugated with BV510, were purchased from BD Biosciences. Staining was performed in RPMI-1640 medium containing 2% FBS. All monoclonal antibodies were added at saturating concentrations, and the cells were incubated for 30 min on ice, washed twice, and analyzed with an LSR II flow cytometer (BD Biosciences) [15, 17].

### Plasma Concentration of ATP

PB was obtained by retro-orbital plexus bleeding into cold microvette EDTA-coated tubes (Sarstedt Inc.). Subsequently, blood was centrifuged at 2000 x g for 20 min at 4 °C to obtain plasma. The ATP levels were measured using the Deproteinizing Sample Preparation kit (BioVision, Milpitas, CA, USA) and the ATP Colorimetric Assay kit (Sigma-Aldrich, St. Louis, MO, USA) according to the manufacturer’s protocol. Absorbance analysis was performed at 570 nm in a microplate reader within 2 h.

### RT-PCR Analysis of Nlrp3 Inflammasome Complex Gene Expression

Total RNA from murine peripheral blood cells was isolated with the RNeasy Kit (Qiagen, Valencia, CA, USA). The RNA was reverse-transcribed with iScript (Bio-Rad). Quantitative assessment of mRNA levels was done by real-time RT-PCR using an ABI 7500 instrument with Power SYBR Green PCR Master Mix reagent. The relative quantity of a target, normalized to the endogenous β2 microglobulin gene as control and relative to a calibrator, is expressed as 2–DDCt (fold difference), where Ct is the threshold cycle, DCt = (Ct of target genes) − (Ct of the endogenous control gene, β-microglobulin), and DDCt = (DCt of samples for the target gene) − (DCt of the calibrator for the target gene). To avoid the possibility of amplifying DNA contamination, uniform amplification of the products was rechecked by analyzing the melting curves of the amplified products (dissociation curves). It was found that the melting temperature (Tm) was 57–60 °C, while the product Tm was at least 10 °C higher than the primer Tm [17]. The following primer pairs were used for analysis:

- β2M
- forward primer: 5′-ATGCTATCCAGAAAACCCCTCAAAT-3′
- reverse primer: 5′-AACTGTGTTACGTAGCAGTTCAGTA-3′
- NLRP3
- forward primer: 5′- ACCAGCCAGAGTGGAATGAC -3′
- reverse primer: 5′- ATGGAGATGCGGGAGAGATA -3′
- CASP1
- forward primer: 5′- GCTTTCTGCTCTTCAACACC -3′
- reverse primer: 5′- AAAATGTCCTCCAAGTCACAAG −3′
- IL-1β
- forward primer: 5′- AGTTGACGGACCCCAAAAG −3′
- reverse primer: 5′- CTTCTCCACAGCCACAATGA -3′
- IL-18
- forward primer: 5′- ACAACTTTGGCCGACTTCAC -3′
- reverse primer: 5′- GTCTGGTCTGGGGTTCACTG -3′
- GSDMD
- forward primer: 5′- CTGGGTCTTGCTAGAAGAATGTGG −3′
- reverse primer: 5′- CTGGCCTAGACTTGACAATAGGAAC -3′
- HMGB1
- forward primer: 5′- GGAGGAGCACAAGAAGAAGC -3′
- reverse primer: 5′- GGGGGATGTAGGTTTTCATTT -3′
- S100A9
- forward primer: 5′- TGGTGGAAGCACAGTTGG −3′
- reverse primer: 5′- CATCAGCATCATACACTCCTCAA -3′
- NR1D1
- forward primer: 5′- TGGCCTCAGGCTTCCACTATG −3
- reverse primer: 5′- CCGTTGCTTCTCTCTCTTGGG -3′
- AIM2
- forward primer: 5′- AGGCAGTGGGAACAAGACAG −3
- reverse primer: 5′- GAAAACTTCCTGACGCCACC -3
- NRLP1a
- forward primer: 5′- AGGTGGAGCTAATGAAGCACA −3′
- reverse primer: 5′- CCATGTTAGAAGAGGGTAAAGAGC -3′
- NRLP1b
- forward primer: 5′- AGAGGTGGAGCTGATGAAGC -3′
- reverse primer: 5′- ACCATGTGGGGTCCAGAGT −3′

### Statistical Analysis

Results are presented as mean ± SD. Statistical analysis of the data was done using Student’s t test for unpaired samples (Excel, Microsoft Corp., Redmond, WA, USA), with a value of *p* ≤ 0.05 considered significant.

## Results

### Diurnal Changes in the PB Level of Extracellular ATP Occur in a Pannexin 1-Dependent Manner

Based on the observation that hypoxia triggers release of ATP into the extracellular space [24, 25], we became interested in whether the ATP level in murine PB changes in a circadian rhythm-dependent manner. We employed the term “zeitgeber; ZT” (translated from the German as “time giver” or “synchronizer”), which was introduced by Jürgen Aschoff, one of the founders of the field of chronobiology, to annotate time points of measurement: ZT1 for 7:00 am, ZT5 for 11:00 am; ZT13 for 7:00 pm, and ZT21 for 3:00 am.

**Fig. 1:**
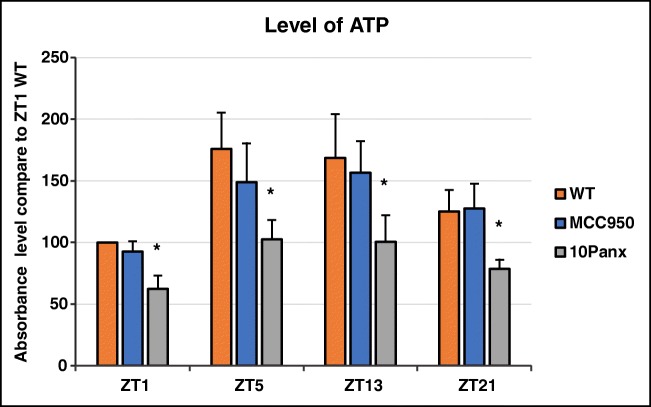
Diurnal changes in the plasma level of ATP. Diurnal activation of ATP in experimental mice was measured by colorimetric assay. The peak level of this extracellular nucleotide in PB occurred between ZT1 (7 AM) and ZT5 (11 AM). Data are pooled from two independent experiments (*n* = 6 mice each) *p ≤ 0.05. As expected, inhibition of the pannexin 1 channel by the 10Panx blocking peptide negatively affected the ATP level in PB. By contrast, MCC950 did not affect the ATP level in PB

As expected (Fig. 1), we found that the ATP level gradually increases in PB plasma, from late night through the morning hours (ZT21–ZT5). Since ATP is released from the stressed cells mainly by pannexin 1 channels, we exposed mice to the pannexin 1-blocking peptide 10Panx, and, as expected, mice exposed to this inhibitor released less ATP into PB. By contrast, no effect on ATP level was seen after exposure of mice to the specific Nlrp3 inflammasome inhibitor MCC950 [22, 23].

### Diurnal Changes in the Expression of Genes from the Nlrp3 Inflammasome Complex

**Fig. 2:**
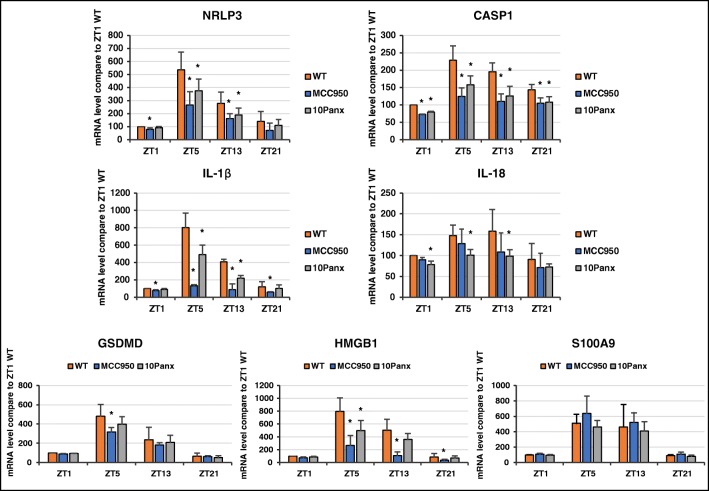
Diurnal changes in the expression of genes involved in the activity of the Nlrp3 inflammasome complex. Expression of the mRNAs for Nlrp3, caspase 1, IL-1β, IL-18, GSDMD, and danger-associated molecular pattern molecules (DAMPs), such as HGMB-1 and S1009A, gradually increased in PB plasma from late night through the morning hours (ZT21–ZT5). Except for S1009A, expression was affected by inhibition of ATP release in a pannexin 1-dependent manner or by exposure to MCC950, a specific inhibitor of the Nlrp3 inflammasome. Data are pooled from two independent experiments (*n* = 6 mice each). *p ≤ 0.05

Extracellular ATP binds to the P2X7 purinergic receptor, which is expressed on the surface of innate immunity cells and activates the Nlrp3 inflammasome [17–21]. Therefore, we evaluated the expression of mRNA for genes involved in the biological effects of the ATP–P2X7 interaction in PB leucocytes. As expected, we observed that the expression of mRNA for Nlrp3, caspase 1, IL-1β, IL-18, gasdermin (GSDMD), and Nlrp3 inflammasome-associated danger-associated molecular pattern molecules (DAMPs), such as high molecular group box 1 (HGMB-1) and S100 calcium-binding protein A9 (S1009A), gradually increase in PB plasma, from late night through the morning hours (ZT21–ZT5, Fig. 2).

Moreover, as expected, expression of these genes was affected by decreasing the release of ATP from cells after exposure of animals to the pannexin 1-blocking peptide 10Panx and in response to a small-molecule inhibitor of the Nlrp3 inflammasome, MCC950. However, in the case of S1009A we did not observe such an inhibitory effect, which suggests that the circadian release of certain DAMPs (e.g., S1009A) may be regulated differently.

### Diurnal Changes in Expression of the Circadian Clock Gene NR1D1 As Well as the Aim2, Nlrp1a, and Nlrp1b Inflammasomes

**Fig. 3:**
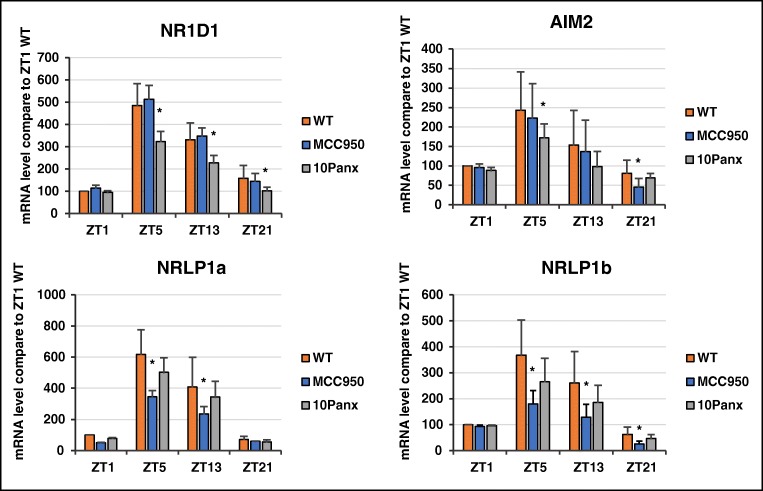
Diurnal changes in expression of NR1D1 and three inflammasomes. Expression of mRNA for NR1D1 as well as the Aim2, Nlrp1a, and Nlrp1b inflammasomes gradually increased in PB plasma from late night through the morning hours (ZT21–ZT5), and except for Aim2 their expression was affected in mice by inhibition of ATP release in a pannexin 1-dependent manner or by exposure to MCC950, a specific inhibitor of the Nlrp3 inflammasome. Data are pooled from two independent experiments (*n* = 6 mice each). *p ≤ 0.05

Nuclear receptor subfamily group D member 1 (NR1D1) protein has recently been described as reducing the circadian activity of the Nlrp3 inflammasome during fulminant hepatitis in mice [25]. Therefore, we selected this circadian clock gene and evaluated the circadian changes in expression of NR1D1 mRNA in peripheral blood granulocytes. We found that diurnal changes in expression of this mRNA were significantly diminished in mice exposed to 10Panx that inhibits secretion of ATP but not to direct Nlrp3 inhibitor MCC950. This finding demonstrated that expression of this gene is regulated differently in hematopoietic cells in response by ATP and Nlrp3 inflammasome activation (Fig. 3). It would be important in the future to evaluate effect of purinergic signaling – Nlrp3 inflammasome on expression of other genes that regulate circadian rhythm [2, 3].

We also evaluated the mRNA expression of other inflammasomes [26] in PB granulocytes and observed diurnal changes in expression of the Aim2, Nlrp1a and Nlrp1b inflammasomes (Fig. 3b).

### Diurnal Changes in the Number of Circulating HSPCs in Murine PB Are Affected by Inhibition of ATP Secretion by the Pannexin 1 Channel and by Inhibition of the Nlrp3 Inflammasome

**Fig. 4:**
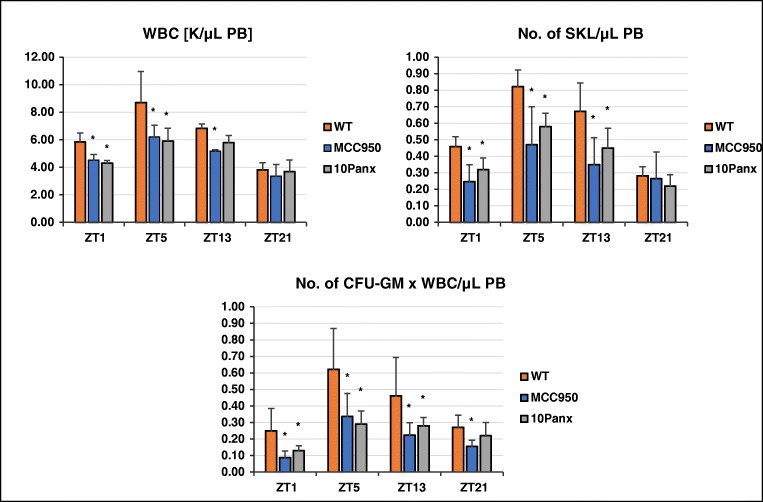
Diurnal changes in the numbers of circulating WBCs, SKL cells, and CFU-GM colonies in mice after inhibition of ATP release in a pannexin 1-dependent manner or after exposure to MCC950, a specific inhibitor of the Nlrp3 inflammasome. The highest number of cells circulating in PB was observed in mice at ZT5 (11 AM). The numbers of these cells were regulated in a pannexin 1- and Nlrp3 inflammasome-dependent manner. Data are pooled from two independent experiments (*n* = 6 mice each). *p ≤ 0.05. All parameters studied in this work were measured at predefined ZT points only

To address the role of ATP–Nlrp3 inflammasome activation in the diurnal release of HSPCs, we analyzed circadian changes in the numbers of WBCs, Sca-1 + c-Kit+Lin– (SKL) cells, and circulating clonogenic CFU-GM in PB (Fig. 4). In control animals we found a circadian pattern in the numbers of cells circulating in PB, with the peak occurring in the late-morning hours (ZT5), which correlated with the highest level of circulating ATP (Fig. 1) and peak activation of the Nlrp3 inflammasome (Fig. 2). However, we found that the diurnal release of cells from BM was decreased in mice exposed to the 10Panx blocking peptide or to MCC950, a specific Nlrp3 inflammasome inhibitor (Fig. 4).

**Fig. 5:**
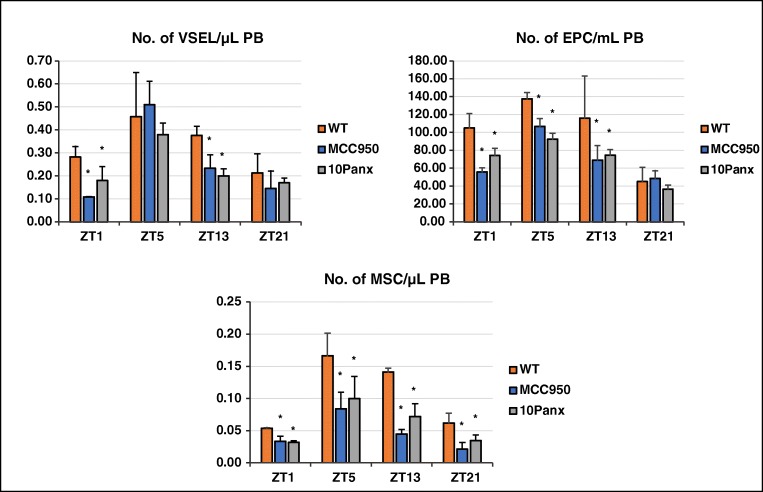
Diurnal changes in the numbers of circulating MSCs, EPCs, and VSELs in mice after inhibition of ATP release in a pannexin 1-dependent manner or after exposure to MCC950, a specific inhibitor of the Nlrp3 inflammasome. The highest number of stem cells circulating in PB was observed in mice at ZT5 (11 AM). The numbers of these cells were regulated in a pannexin 1- and Nlrp3 inflammasome-dependent manner. Data are pooled from two independent experiments (*n* = 6 mice each). *p ≤ 0.05. All parameters studied in this work were measured at predefined ZT points only

In parallel, we evaluated changes in circadian circulation of other BM-residing stem cell/progenitor cells, including mesenchymal stromal cells (MSCs), endothelial progenitor cells (EPCs), and very small embryonic-like stem cells (VSELs, Fig. 5). As with HSPCs (Fig. 4) the peaks occurred in the late morning hours (ZT5), which correlated with the highest level of circulating ATP (Fig. 1) and peak activation of the Nlrp3 inflammasome (Fig. 2). Moreover, the diurnal release of these cells from BM into PB was diminished in mice exposed to the 10Panx blocking peptide for the pannexin 1 channel or to MCC950, a specific inhibitor of the Nlrp3 inflammasome (Fig. 5).

These results show that all types of BM-residing stem cells, hematopoietic and non-hematopoietic, show a circadian rhythm in their circulation in an ATP–Nlrp3 inflammasome-dependent manner.

## Discussion

The salient observation of this study is that diurnal changes in the ATP level in PB activate the Nlrp3 inflammasome for release of stem cells from BM into PB. Thus, we show for the first time the presence of an interplay between purinergic signaling and innate immunity in orchestrating circadian changes in the numbers of circulating hematopoietic stem/progenitor cells (HSPCs) and non-hematopoietic stem/progenitor cells (MSCs, EPCs, VSELs) in PB.

The circadian changes in the number of circulating HSPCs in PB were the subject of several excellent studies showing a peak in the early morning hours and a nadir at night [5–13]. This phenomenon has been attributed to diurnal changes in tonus of the vegetative nerves that innervate BM tissue as a result of external light and dark cycles [4, 9, 10]. Since the level of catecholamines increases in PB in the morning hours, it has been proposed that circadian changes in their level determine circadian changes in the number of circulating HSPCs. Supporting this interpretation, UDP-galactose:ceramide galactosyltransferase-deficient mice, which exhibit aberrant nerve conduction, do not release norepinephrine (NE) into the BM microenvironment in the morning in response to external light exposure and do not efficiently mobilize HSPCs [4, 9].

Recently, another group has demonstrated that light-induced norepinephrine and TNF secretion augments HSPC differentiation and increases vascular permeability, which releases HSPCs into PB in a circadian rhythm-dependent manner [6]. At the same time, darkness-induced TNF increases melatonin secretion, which drives renewal of HSPCs and diminishes vascular permeability. These findings demonstrated that daily light- and darkness-induced bursts of norepinephrine, TNF, and melatonin within the BM microenvironment are essential for synchronized diurnal release of HSPCs into PB [6].

Our team reported that activation and crosstalk between the complement cascade (ComC), the coagulation cascade (CoaC), and the fibrinolytic cascade (FibC) is essential for circadian release of HSPCs from BM into PB, as all these proteolytic cascades show circadian activation late at night or in the early morning hours due to deep-sleep hypoxia [7, 12, 13]. Specifically, diurnal activation of the ComC, CoaC, and FibC in WT animals in the early morning hours precedes the release of HSPCs [13, 27]. By contrast, we did not observe diurnal changes in the number of circulating cells in PB in mice deficient for the ComC protein C5, which is pivotal in executing ComC-dependent release of HSPCs from BM into PB [13].

As recently acknowledged, sphingosine-1-phosphate (S1P) is an important chemoattractant in PB and is responsible for egress of HSPCs from their BM niches [14]. Interestingly, we also demonstrated that the plasma level of S1P fluctuates in patients in a circadian rhythm-dependent manner, with the peak in the early morning hours and the nadir late at night [8]. This fluctuation in S1P level can be explained by activation of the ComC due to hypoxia during deep sleep, which by generating C5b–C9 (also known as membrane attack complex, MAC) releases S1P from red blood cells and activated platelets [8].

Based on the observation that purinergic signaling involving extracellular ATP mediates crosstalk with the innate immunity system in the mobilization of HSPCs and the fact that there are diurnal changes in ATP released from cells, we became interested in whether circadian changes in ATP level in PB promote mobilization of HSPCs. We found that the ATP level in PB undergoes diurnal fluctuations and that this occurs in a pannexin 1 channel-dependent manner. Moreover, since during pharmacological mobilization ATP enhances egress of HSPCs from BM into PB after binding to the inotropic purinergic P2X7 receptor and activation of Nlrp3 inflammasome (as reported by us recently), we inhibited the Nlrp3 inflammasome by employing the specific small-molecule inhibitor MCC950.

Inhibition of the Nlrp3 inflammasome resulted in a dampening of diurnal changes in the number of circulating HSPCs. Nevertheless, this dampening was not complete, which suggests that other mechanisms may be involved, such as compensatory activation of other members of the inflammasome family, which, as reported here, also become activated in a diurnal manner. However, this question requires further study. On the other hand, despite the fact that MCC950 is a specific inhibitor of the Nlrp3 inflammasome [22, 23], future circadian rhythm studies in Nlrp3-KO mice may reveal a more profound decrease in the diurnal level of circulating stem cells, similar to that observed in C5-KO mice [13]. Specifically, activation of the Nlrp3 inflammasome leads to activation of the ComC, and C5 cleavage fragments orchestrate egress of HSPCs into PB [17, 21]. Therefore, circadian rhythm studies in Nrlp3-KO mice will provide better insight into the importance of the ATP–Nlrp3 inflammasome axis in diurnal activation of the ComC.

Interestingly, in contrast to our previous report [13], we observed clear evidence of circadian rhythms in the circulation of other stem/progenitor cells, such as MSCs, EPCs, and VSELs. These inconsistencies are most likely explained by differences in the mice colonies maintained in different laboratories that were employed at different time in the past and current experiments.

We are aware that these studies performed in mice need to be verified in patients. There are obvious differences in circadian rhythms in mice and humans, based on the fact that mice are nocturnal animals and their active and rest phases are out of phase with humans. Moreover, there may be differences in regulation of circadian rhythms between murine strains, and we expect sex- and age-dependent differences. Moreover, while the abovementioned catecholamines are involved in sympatho-adrenomedullary regulation of cardiovascular, respiratory, and metabolic functions, extensive study in humans has demonstrated that the morning increase in the level of catecholamines is mainly in response to changes in activity and posture rather than to an endogenous circadian surge of plasma catecholamines due to the onset of daylight [28, 29]. This latter finding may explain discrepancies in the literature between the apparent role of catecholamines in the release of HSPCs in humans versus mice. In support of this possibility, it has been reported that in normal human HSPC donors receiving noradrenaline (NE) reuptake inhibitors (NRI) for depression or β2-blockers for hypertension mobilization is induced in a similar manner as normal controls and was neither enhanced by NRI administration nor suppressed in the presence of β2-blockers [30]. The potential role of catecholamines in regulating circadian release of HSPCs from BM into PB has also been challenged in humans: while infusion of beta receptor agonists increased the number of circulating leucocytes (most likely by release from the marginal pool in the vasculature), it did not affect the number of circulating HSPCs. Finally, one has to consider whether experimental murine models based on environmental stimuli such as exposure to light/darkness are related to internally encoded circadian rhythms that occur without exposure to external stimuli [1].

The link described here between purinergic signaling and innate immunity also has important implications for better understanding the field of chrono-immunology. Several molecular clock proteins (e.g., BMAL1 and CLOCK) have been shown to directly affect transcription of several genes involved in immune responses. Moreover, perturbations of circadian rhythms in humans caused by shift work or jet lag may negatively affect immune responses in humans [1, 2, 7, 28, 31].

In conclusion, our results demonstrate for the first time the existence of a link between purinergic signaling [32, 33] and innate immunity [17, 21] in regulating the circadian circulation of stem cells in PB. They also provide a better understanding of the phenomenon of chrono-immunology. However, we are aware that results obtained in a murine model require verification in humans.
